# Design and rationale of the EFFORTII project: a multicentric randomised-controlled trial on the impact of continued nutritional therapy at hospital discharge

**DOI:** 10.1136/bmjopen-2025-115456

**Published:** 2026-03-18

**Authors:** Carla Wunderle, Pascal Tribolet, Nina Kaegi-Braun, Valerie Haller, Robert Escher, Drahomir Aujesky, Gisele Trennepohl Da Costa Heinen, Michael Brändle, Thomas Bregenzer, Christoph Henzen, Thomas Zehnder, Susanne Schait, Christina Gassmann, Maja Dorfschmid, María D Ballesteros-Pomar, Cristina Cuerda, Rosa Burgos, Daniel De Luis, Gabriel Olveira, Leocardio Rodriguez-Mañas, Zeno Stanga, Beat Mueller, Philipp Schuetz

**Affiliations:** 1 Medical University Department, Division of General Internal and Emergency Medicine, Kantonsspital Aarau AG, Aarau, Switzerland; 2 Department of Health Professions, Bern University of Applied Sciences, Bern, Switzerland; 3 Faculty of Life Sciences, University of Vienna, Vienna, Austria; 4 Department of Medicine Huddinge, Karolinska Institute, Stockholm, Sweden; 5 Medical University Department, Kantonsspital Aarau, Aarau, Switzerland; 6 Department of Nutritional Science, Justus Liebig University Giessen, Giessen, Germany; 7 Department of Medicine, Spital Emmental, Burgdorf, Switzerland; 8 Division of Diabetes, Endocrinology, Nutritional Medicine, and Metabolism, Bern University Hospital, Bern, Switzerland; 9 Department of Endocrinology, Kantonsspital Münsterlingen, Münsterlingen, Switzerland; 10 Department of General Internal Medicine / Family Medicine and Emergency Medicine, HOCH Health Eastern Switzerland, St. Gallen, Switzerland; 11 Medical Department, Spital Lachen AG, Lachen, Switzerland; 12 Medical Department, Luzerner Kantonsspital, Lucerne, Switzerland; 13 Medical Department, Spital Thun, Thun, Switzerland; 14 Department of Anesthesiology and Critical Care Medicine, Klinik Hirslanden, Zürich, Switzerland; 15 Department of Nursing and Allied Health Care Professions, University Hospital Zurich, Zürich, Switzerland; 16 Clinic for Visceral, Thoracic, Vascular Surgery, and Angiology, Stadtspital Zürich Triemli, Zürich, Switzerland; 17 Department of Endocrinology and Nutrition, Complejo Asistencial Universitario de León, Leon, Spain; 18 Departamento de Medicina, Hospital General Universitario Gregorio Marañón, Madrid, Spain; 19 Nutritional Support Unit, University Hospital Vall d’Hebron, Madrid, Spain; 20 Servicio de Endocrinología y Nutrición, Hospital Clínico Universitario de Valladolid and Health Research Institute of Valladolid (IBioVALL), Valladolid, Spain; 21 Centro de Investigación Biomedica en Red (CIBEROBN) de la Obesidad y Nutrición, Universidad de Valladolid, Valladolid, Spain; 22 Servicio de Endocrinología y Nutrición, ospital Regional Universitario e instituto de investigación biomédica de Málaga, Malaga, Spain; 23 Departamento de Medicina y Dermatología, Universidad de Málaga, Málaga, Spain; 24 Centro de Investigación Biomédica en Red (CIBER) de Diabetes y Enfermedades Metabólicas Asociadas, Instituto de Salud Carlos III, Málaga, Spain; 25 Centro de Investigación Biomédica en Red sobre Fragilidad y Envejecimiento Saludable (CIBERFES), Instituto de Salud Carlos III, Madrid, Spain; 26 Servicio de Geriatría, Hospital Universitario de Getafe, Getafe, Spain; 27 Division of Diabetes, Endocrinology, Nutritional Medicine, and Metabolism, Inselspital Universitatsspital Bern, Bern, Switzerland; 28 Medical University Department, Kantonsspital Aarau AG, Aarau, Switzerland; 29 Medical Faculty, University of Basel, Basel, Switzerland

**Keywords:** Randomized Controlled Trial, NUTRITION & DIETETICS, Treatment Outcome, INTERNAL MEDICINE, Nutritional support

## Abstract

**Introduction:**

Malnutrition is a highly prevalent chronic condition that contributes to higher morbidity and mortality in patients with multiple comorbidities. While positive effects of nutritional therapy in the in-hospital setting have recently been demonstrated, the benefits of long-term nutritional therapy after hospital discharge remain uncertain. Herein, we outline the design and rationale of the EFFORTII trial, the largest nutritional trial to date to assess the effects of continued nutritional support after hospital discharge in medical patients, with particular attention to key design decisions regarding nutritional strategy, patient selection criteria and study endpoints.

**Methods and analysis:**

The *Effect of Continued Nutritional Support at Hospital Discharge on Mortality, Frailty, Functional Outcomes and Recovery* (EFFORTII) is an investigator-initiated, non-commercial randomised controlled trial designed to evaluate whether ongoing, individualised nutritional therapy after hospital discharge—targeted to meet specific energy and protein requirements—offers a cost-effective approach to lowering mortality, minimising complications and maintaining functional status compared with standard care. Eligible participants are adult, chronically ill medical inpatients at risk of malnutrition. Patients in the intervention group receive individualised nutritional therapy delivered by an experienced dietitian through a combination of telemedicine and in-person consultations. The intervention aims to meet personalised nutritional targets, supported by a trained dietitian. Control group patients receive nutritional counselling at discharge, but no structured nutritional management during follow-up. We designed the trial as an event-driven trial with a target of 247 mortality events (primary endpoint), which will be assessed over approximately 5 years until event-driven endpoint is met. The minimum total sample size is at least 802 participants, based on the assumed treatment HR of 0.70. The main trial is enrolling patients across multiple sites in Switzerland. During the trial, additional sites in Spain joined the study, and their data will be analysed using a patient-level pooled approach.

**Ethics and dissemination:**

This study involves human participants and was first granted ethical approval by the Ethics Committee Northwest- and Central Switzerland and then by all participating local ethics committees. Written informed consent will be obtained from all participants. Findings will be disseminated in peer-reviewed journals and academic conferences.

**Trial registration number:**

NCT04926597.

**STRENGTH AND LIMITATIONS OF THIS STUDY**

- This trial will be the largest outpatient trial conducted to date, enrolling a diverse population of polymorbid medical patients, thereby enhancing the generalisability of the findings.
- The multicentre design and long follow-up period improve external validity and increase the robustness of the results.
- The primary outcome will be assessed in a blinded manner, minimising bias.
- The absence of blinding for study participants and caregivers may introduce bias.
- The long study duration may result in reduced adherence over time.

## Introduction

 Disease-related malnutrition, hereafter malnutrition, in hospitalised adult medical patients is a complex syndrome associated with substantially high morbidity, disability, both short- and long-term mortality, delayed recovery and increased healthcare costs compared with individuals without malnutrition.^1^,^3^ Multiple factors increase the risk of malnutrition in chronically ill polymorbid patients. First, anorexia, as part of the physiological response to acute illness or metabolic stress, increases the risk of significant energy and protein deficiencies in polymorbid patients.^4 5^ In combination with immobilisation and a pronounced inflammatory (ie, interleukin 6-mediated) and endocrine stress response (eg, low levels of sex hormones, increased levels of steroid hormones), these nutritional deficits contribute to muscle wasting and progressive deterioration of metabolic and functional status.^6^,^10^ As a result, malnutrition affects up to 30%–50% of medical inpatients and is a strong and independent long-term risk factor for mortality, rehospitalisations and functional decline in this population.^11^,^13^ Importantly, since malnutrition is a chronic condition, its risks often persist after hospital discharge, exposing patients to long-term morbidity and mortality.^14 15^ In fact, data from the Effect of Early Nutritional Support on Frailty, Functional Outcomes and Recovery of Malnourished Medical Inpatients (EFFORT) trial showed that when nutritional support was discontinued at hospital discharge, mortality among malnourished medical patients was approximately 10% 1 month after discharge, rising to 20% at 6 months and 60% after 3 years of follow-up.^14^,^16^ Even though mainly based on observational data, we assume that an important part of this excess mortality risk is directly attributable to malnutrition.^7 11^ Particularly, because current evidence from clinical trials indicates that malnutrition is, at least partly, a modifiable risk factor that can be mitigated through individualised nutritional therapy aimed at reaching nutritional targets.^16^,^18^

### Current evidence from randomised trials

**Figure 1:**
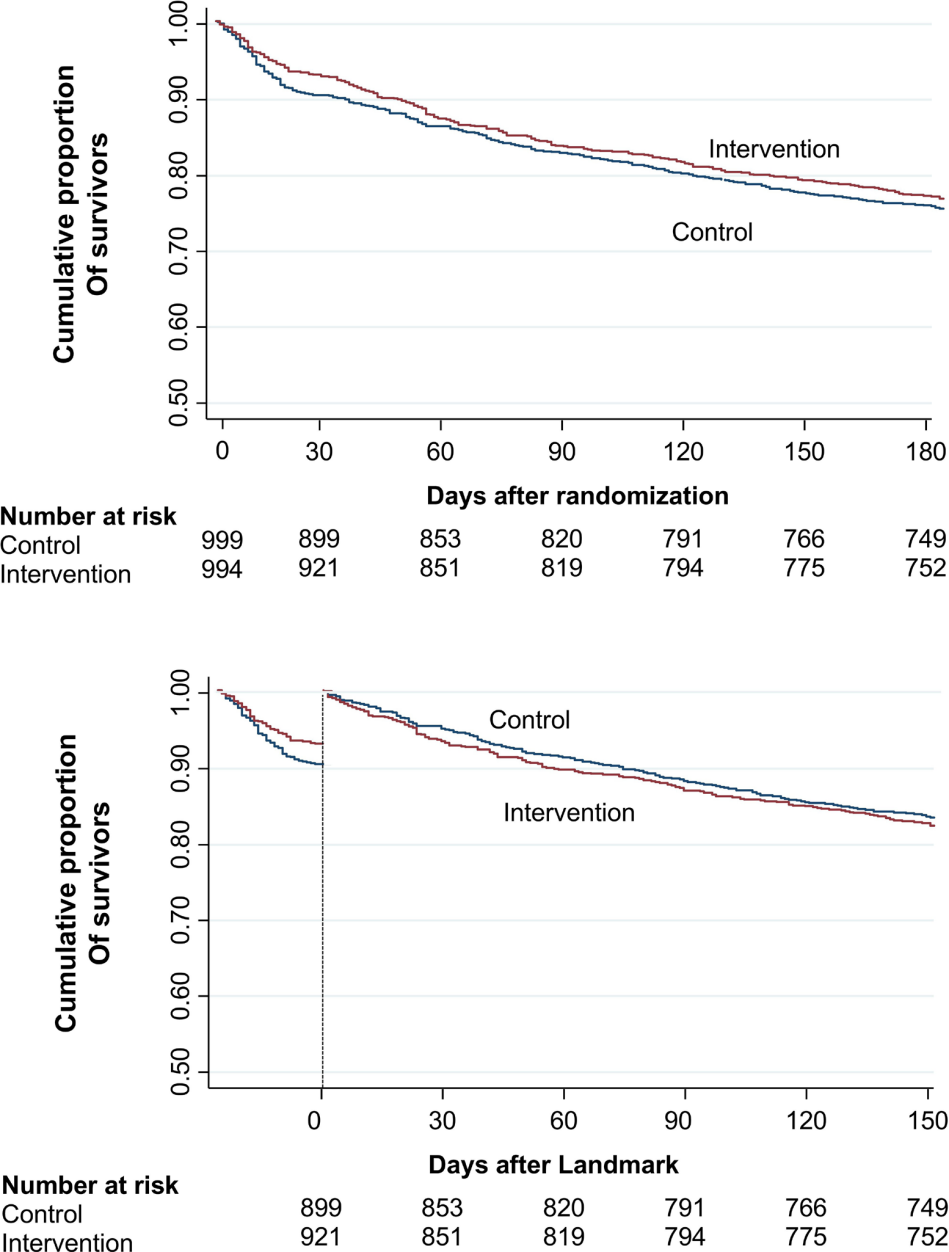
Kaplan-Meier estimates of the cumulative incidence of all-cause mortality (**A**) and landmark analysis (**B**).^20^ Panel A shows the Kaplan-Meier curves for the time to death within 6 months (p log rank 0.45). Panel B shows the landmark analysis of time to death after 30 days (p log rank 0.52).

Recent clinical trials on nutritional therapy in medical inpatients have advanced our understanding of disease processes, showing that early and structured nutritional interventions can improve outcomes and lower mortality—at least in the short term.^1^ A 2019 systematic review and meta-analysis included 27 trials comprising 6803 patients, reported that nutritional therapy provided during the hospital stay was associated with a 25% reduction in both mortality and non-elective hospital readmissions.^19^ Among these trials, EFFORT was the largest trial with over 2000 patients and compared the effects of individualised nutritional therapy to reach energy and protein targets compared with usual care in eight Swiss hospitals.^16^ In this trial, the nutritional intervention was highly effective in lowering the risk for mortality with a number needed to treat (NNT) of 37. A similar positive effect on the risk of mortality (NNT=20) was also found in the US-based, 652 patients NOURISH (Nutrition effect On Unplanned ReadmIssions and Survival in Hospitalized patients) trial^18^ and other more recent trials. Importantly, most trials included in the above-mentioned meta-analysis examined only in-hospital nutritional interventions. Findings from the EFFORT study, which stopped the nutritional intervention at discharge, suggest that this may be insufficient, as nutritional therapy showed no significant long-term effect on mortality in a secondary analysis. Specifically, individualised nutritional therapy during hospitalisation significantly lowered short-term mortality (adjusted OR 0.79 (95% CI 0.64 to 0.97)) but did not confer a lasting benefit on 180-day mortality (adjusted HR 0.90 (95% CI 0.76 to 1.08)).^20^ Despite these results, the study confirmed that this population remains highly vulnerable, with persistently elevated mortality rates (figure 1).

### Rationale of the study and overall aim

Current clinical practice guidelines, including those from the European Society for Clinical Nutrition and Metabolism (ESPEN)^21^ and the American Society for Parenteral and Enteral Nutrition (ASPEN),^22^ provide only limited recommendations for the use of nutritional therapy in the outpatient setting for patients at nutritional risk, despite the physio-pathological rationale for addressing this risk factor. This lack of recommendation is mainly due to the paucity of high-quality evidence proving the positive effects of outpatient nutritional therapy. Nevertheless, at least one meta-analysis that included randomised controlled trials (RCTs) conducted in outpatient settings found that nutritional interventions were linked to a significant reduction in all-cause mortality up to 12 months (OR of 14 RCTs involving 2438 participants 0.63 (95% CI 0.48 to 0.84)).^23^ However, studies included in this analysis had mostly moderate trial quality and low sample sizes. Therefore, a large conclusive intervention trial is urgently needed to determine whether medical patients at nutritional risk show a sustained benefit from long-term nutritional therapy including several interventions led by a dietitian, for example, high protein-high calorie oral nutritional supplements after hospital discharge. In addition, we want to elucidate the mechanisms by which nutritional therapy affects disease progression from a mechanistic physio-pathological perspective. Furthermore, we would like to clarify the indications for which nutritional therapy is cost-effective by incorporating pharmacoeconomic research.

## Methods

### Study design and setting

**Figure 2:**
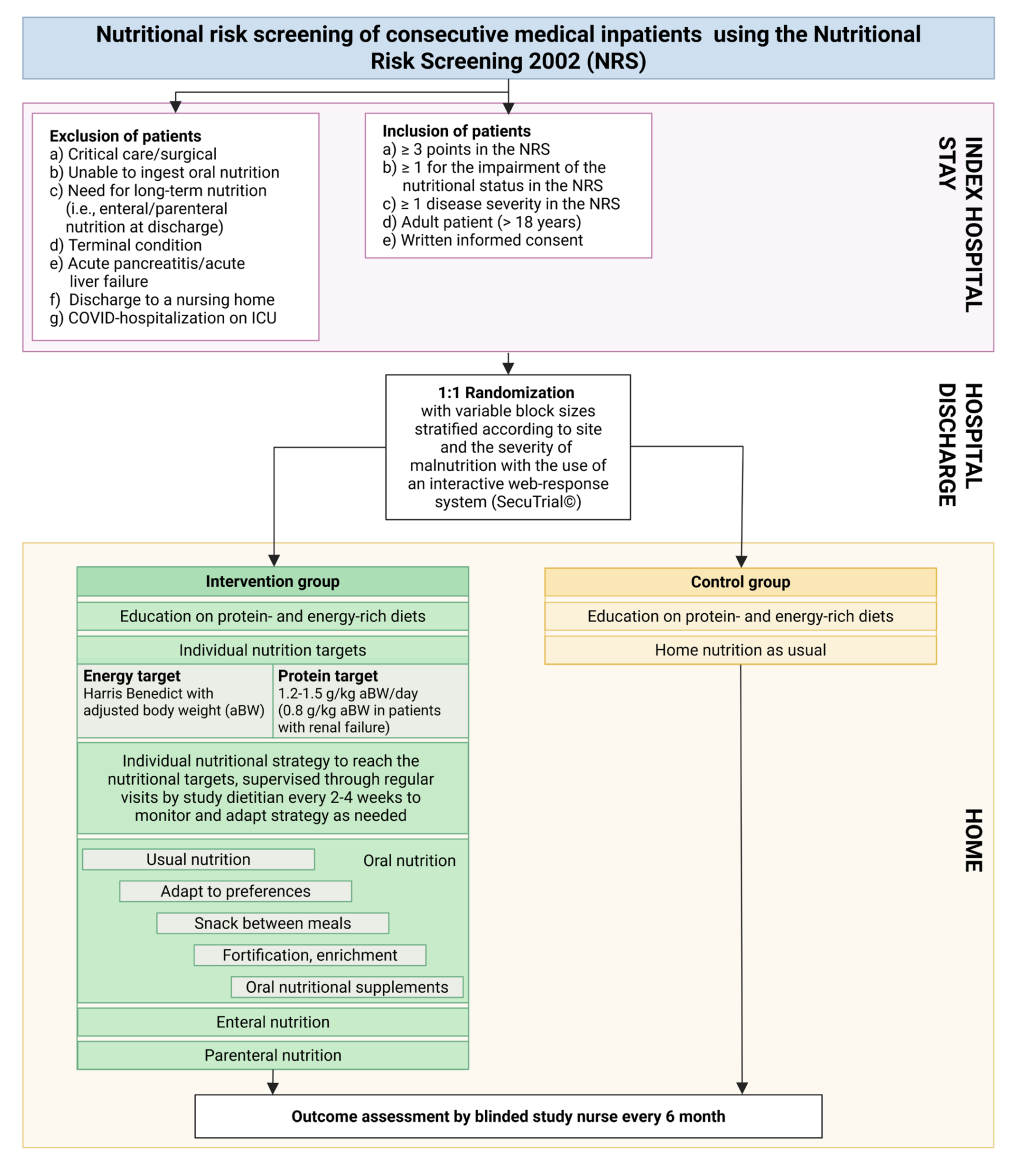
Study flow. ICU, intensive care unit.

We conduct an investigator-initiated, pragmatic, single-blinded, RCT. The overall aim is to test the hypothesis that sustained post-discharge nutritional therapy to reach individual energy and protein targets compared with usual care in medical patients at nutritional risk is a cost-effective strategy to reduce mortality, prevent complications and a decline in functional capacity. Figure 2 shows the principal patient flow from screening through inclusion, randomisation and treatment to the assessment of patient outcomes. The steering committee is described in table 1.

**Table 1:** List of steering committee members

Index	Member
1	Philipp Schuetz
2	Pascal Tribolet
3	Carla Wunderle
4	Nina Kaegi-Braun
5	Maria D Ballesteros-Pomar
6	Zeno Stanga
7	Beat Mueller

The trial started recruitment in 2021 among several sites in Switzerland including the Kantonsspital Aarau, Inselspital Bern, Kantonsspital Luzern, Kantonsspital St.Gallen, Kantonsspital Münsterlingen, Spital Thun, Spital Burgdorf, Spital Lachen, Klinik Hirslanden Zürich, Stadtspital and Universitätsspital Zürich.

While the main trial is recruiting patients across several sites in Switzerland, additional Spanish sites joined the trial in 2024 with the exact same study protocol when the Swiss sites had already included 80% of planned patients. Data from the Spanish sites will be evaluated separately from the main trial through a patient-level pooled analysis. Specifically, the Spanish sites include the Complejo Asistencial Universitario de León, Hospital General Universitario Gregorio Marañón, Hospital Universitario Vall d’Hebron, Hospital Clínico Universitario de Valladolid, Hospital Universitario Regional de Málaga and Hospital Universitario de Getafe. Table 2 contains a full list of EFFORTII study team members.

**Table 2:** Full list of EFFORTII study team

	Site	Investigators
*Switzerland*		
1	Kantonsspital Aarau	Philipp Schuetz, Carla Wunderle, Pascal Tribolet, Nina Kaegi-Braun, Cornelia Julien, Mirjam Martensson, Manuela Deiss, Zeljka Caldara, Valerie Haller
2	Spital Zofingen	Nina Kaegi-Braun, Jolanda Siegenthaler, Sandra Weibel
3	Spital Emmental	Robert Escher, Bernard Chappuis, Marzia Stämpfli, Livia Galli, Anne Etienne, Tamara Antener
4	Inselspital Bern	Drahomir Aujesky, Zeno Stanga, Christa Dürig, Andrea Bovisi
5	Kantonsspital Münsterlingen	Gisele Trennepohl da Costa Heinen, Vojtech Pavlicek, Sanja Sauter
6	Kantonsspital St. Gallen	Michael Brändle, Sarah Sigrist, Sabrina Rüegsegger, Jana Schönenberger, Carmen Benz, Elisabeth Huemer, Patricia Christl, Anina Schönholzer, Raffaela Giulia Martinetti, Livia Bont, Alexandra Beier, Simone Thürlemann
7	Spital Lachen	Thomas Bregenzer, Bruno Schiesser, Seraina Carisch, Nicole Blöchlinger, Selina De Martin
8	Luzerner Kantonsspital	Christoph Henzen, Tullia Lacher, Alessia Priuli, Lena Stalder
9	Spital Thun	Thomas Zehnder, Katrin Montanaro, Celina Locher, Jana Gerber, Carolyn Schmutz, Julia Dietrich, Lisa Gerber
10	Klinik Hirslanden Zürich	Susanne Schait, Patrizia Christen, Reto Stocker
11	Universitätsspital Zürich	Philipp Gerber, Christina Gassmann, Alessia Marino, Esther Haller
12	Stadtspital Zürich Triemli	Maja Dorfschmid, Janna Schraven, Patricia Brandenberger, Nadine Zulliger, Julia Sturzenegger
*Spain*		
1	Complejo Asistencial Universitario de León	María D. Ballesteros Pomar, Diana García Sastre, Maria López Melgar, Elena González Arnáiz, María García Duque
2	Hospital General Universitario Gregorio Marañón, Madrid	Cristina Cuerda, Atocha Bielza, Tamara Hernández, Beatriz Rodríguez, María Luisa Carrascal
3	Hospital Universitari Vall Hebron, Barcelona	Rosa Burgos, Fernanda Mucarzel, Raúl Cartiel
4	Hospital Clínico Universitario de Valladolid	Daniel Antonio de Luis Roman, Olatz Izaola Jauregui, Mario Saavedra Vasquez, Paloma Perez Lopez, Emilia Gomez Hoyos, Juan Jose Lopez Gomez
5	Hospital Universitario Regional de Málaga	Gabriel Olveira, Marina Padial-Barranco, Montserrat Gonzalo-Marin, Carmen Bautista-Recio, Rosario Vallejo-Mora
6	Hospital Universitario de Getafe	Leocadio Rodriguez-Mañas**,** Blanca Alfonso López, Luz Rodríguez-Piñero, Laura Pedraza Sepúlveda, Alejandro Alvarez-Bustos

### Patient eligibility for inclusion and recruitment

During hospitalisation we include adult (age ≥18 years) polymorbid, medical inpatients at nutritional risk (Nutritional Risk Screening 2002 score (NRS): total score ≥3 points).^24^

Participants who meet the following inclusion criteria are eligible for the study:

- Informed consent as documented by signature (online supplemental figure 1).
- Adult (age ≥18 years), medical inpatients.
- Nutritional risk screening using the NRS: total score ≥3 points consisting of ≥1 points for impairment of the nutritional status (weight loss >5% in 3 or 2 months or food intake of 50%–75% or 25%–50% in the last week before hospital admission) plus ≥1 for the severity of the disease (ie, cancer, chronic kidney disease, chronic heart failure, chronic obstructive pulmonary disease (COPD)) and other chronic diseases according to the definition of the National Center for Chronic Disease Prevention and Health Promotion: Chronic diseases are defined broadly as conditions that last 1 year or more and require ongoing medical attention or limit activities of daily living or both.

Excluded are patients who meet the following criteria:

- After surgery.
- Unable to ingest oral nutrition.
- Need for long-term nutrition.
- Terminal condition.
- Acute pancreatitis or acute liver failure.
- Patients discharged to a nursing home.
- Patients unlikely to comply with nutritional treatment (eg, dementia).

Initially, patients with severe COVID-19 infections were excluded from the study due to ethical considerations. However, this criterion was revised in 2023 following the end of the COVID-19 pandemic.

For patient recruitment, all participating hospitals actively screen patients at risk of malnutrition, using the NRS. For all eligible patients, the study staff explains to each participant the nature of the study, its purpose, the procedures involved, the expected duration, the potential risks and benefits and any discomfort it may cause. Each participant is informed that participation in the study is voluntary, that they may withdraw from the study at any time and that withdrawal of consent does not affect their subsequent medical assistance and treatment. Patients have at least 24 hours to consider trial participation. Damage caused by the trial will be covered by the hospital’s liability insurance for Category A studies.

### Data collected at study entry

After trial inclusion, each patient receives a structured systematic medical and nutritional assessment by the study dietitian and study physician including:

- Sociodemographics and anthropometrics (eg, age, sex, weight and height for calculation of body mass index (BMI), food insecurity survey).
- Detailed medical history including information about comorbidities, drug use and results of blood work-up as available from routine care (eg, kidney function based on estimated glomerular filtration rate, electrolytes, albumin, levels of trace elements and vitamins, results of endocrine function).
- Current nutritional intake regarding protein and energy estimated from the patient’s medical charts (as available) or using a structured 24-hour recall.
- Baseline nutritional risk based on risk scores (eg, NRS, Mini Nutritional Assessment or similar).
- Baseline muscle mass (calf circumference).
- Basal metabolic rate/energy requirements calculated based on the Harris-Benedict equation.
- Baseline muscle strength (hand grip dynamometry).
- Baseline body composition (bioelectrical impedance analysis (BIA).
- Baseline functional status (Barthel Index).
- Baseline quality of life (European Quality of Life 5 Dimensions (EQ-5D) Index and EQ-5D visual analogue scale (VAS)).

We also systematically collect blood samples at the main site (Cantonal Hospital Aarau) on study enrolment (day 0) for later batch analyses of blood biomarkers (ancillary project).

### Monitoring

Systematic monitoring will be conducted in accordance with the monitoring plan, which includes an initial visit, an internal monitoring visit after 2–10 patients have been enrolled, an annual internal monitoring visit and a final visit. Monitoring includes review of the screening strategy, informed consent, inclusion/exclusion criteria, primary endpoint, secondary endpoint, serious adverse event (SAE), study documents and data set.

### Randomisation

After patient enrolment, a member of the study team randomises patients 1:1 into the intervention or control group at a time point close to hospital discharge according to a pre-specified, computer-generated, web-based randomisation scheme using the centralised secuTrial website. The randomisation is stratified for site and NRS total score.

### Study endpoints

All patients in both groups are contacted every 6 months by structured phone calls from a blinded study nurse or dietitian to assess primary and secondary endpoints until the patient reaches the primary endpoint, drops out or until the study is terminated. If necessary, information on patient mortality, rehospitalisations or major complications during the follow-up period is confirmed through family members or the patient’s primary care physician.

The primary endpoint is defined as the time from trial inclusion to death from any cause (ie, all-cause mortality). Secondary endpoints are defined as:

- Time to non-elective hospital readmission after discharge from the index hospital stay.
- Time to the first major complication including death, bacterial infection with need for antibiotic treatment, major cardiovascular event (ie, stroke, intracranial bleeding, cardiac arrest, myocardial infarction) or pulmonary embolism, acute renal failure, gastro-intestinal events (including haemorrhage, intestinal perforation, acute pancreatitis).
- Changes in functional status measured by the Barthel’s index (scores range from 0 to 100, with higher scores indicating better functional status).^25^
- Changes in quality of life measured with the EQ-5D index (German Version, EQ-5D index values range from 0 to 1, with higher scores indicating better quality of life) including the VAS (EQ-5D VAS) (scores range from 0 to 100, with higher scores indicating better health status).

We will follow participants until the end of the study, ie, until approximately 247 mortality events have occurred. We expect a maximum follow-up period of 5 years for patients enrolled at the start of the study. In addition to blinded telephone follow-up examinations, the unblinded study dietitian conducts home visits or in-clinic at 3, 6 and 12 months for all patients to evaluate the study-specific nutritional outcomes.

- Body weight.
- Calf circumference to assess muscle mass.
- Bioelectrical impedance analysis to assess body composition (fat-free mass, fat mass).
- Hand grip by means of handgrip strength through dynamometry (Jamar Hydraulic Hand Dynamometer) to assess muscle strength.^26^

A specific process has been defined to identify and report certain SAEs that are not collected as study endpoints. The data is collected centrally via SecuTrial (including queries, rules and feedback for the input fields, so that typos, etc, can be detected and corrected immediately). A list of protocol deviations will be maintained.

### Nutritional treatment of intervention group and control group patients

**Figure 3:**
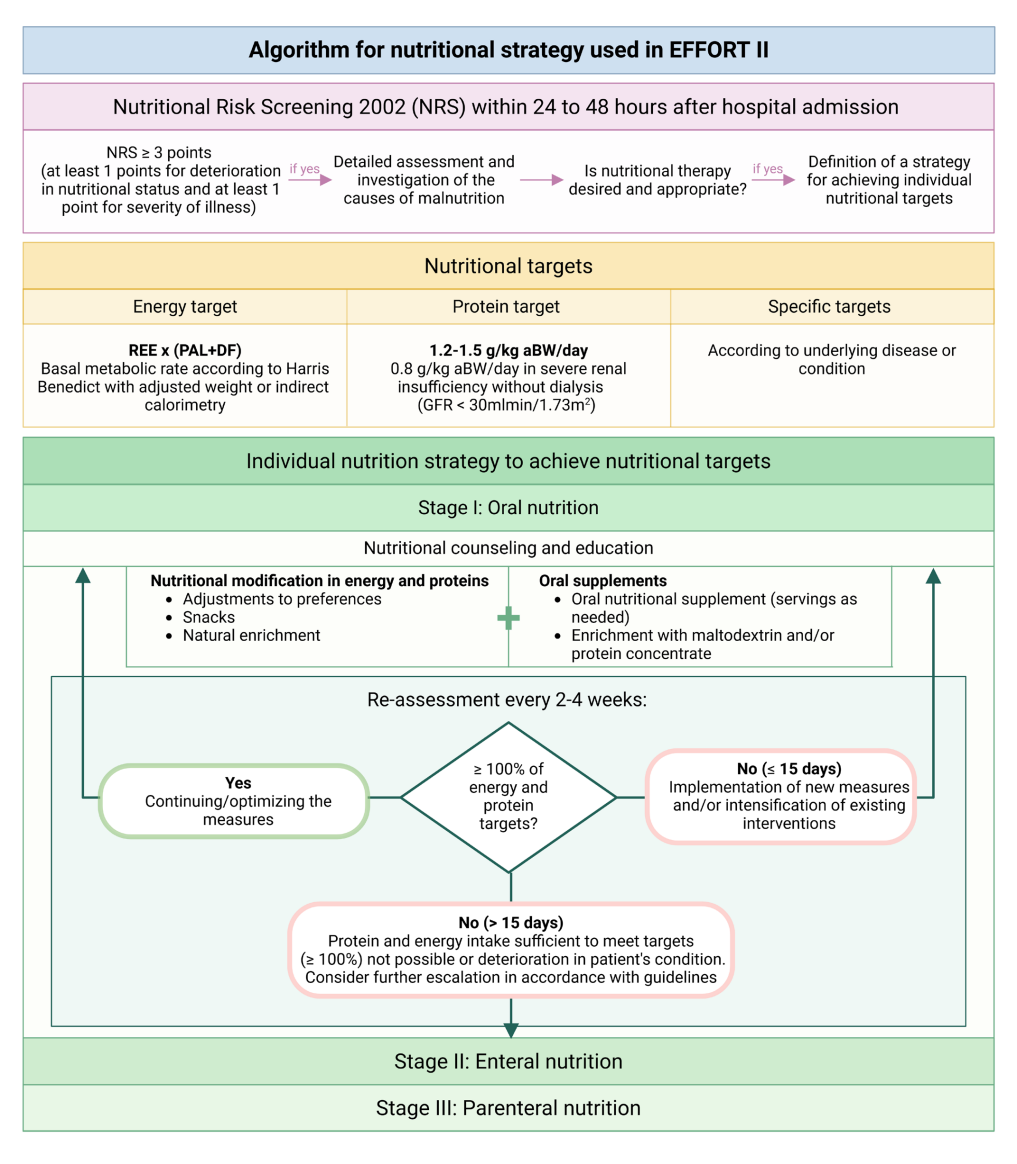
Algorithm for nutritional strategy used in EFFORTII. aBW, adjusted body weight; DF, disease factor; GFR, glomerular filtration rate; PAL, physical activity level; REE, resting energy expenditure.

We have previously developed international nutritional guidelines focusing on the optimal nutritional therapy for medical patients in the inpatient setting.^2127^,^29^ The nutritional intervention is supported by a web-based nutritional management system (NutriOrg) developed by the EFFORT research team, which enables evidence-based, structured nutrition management. NutriOrg is a previously unpublished system that combines the previously publicly available tools from clinicalnutrition.science.^30^ This trial focuses on the post-discharge outpatient setting. Accordingly, the nutritional guidelines for patients in the intervention group are based on similar principles but adapted to enhance practicality and long-term adherence. The overall goal is to cover nutritional requirements regarding daily energy and protein intake. For each intervention group patient, an unblinded, trained and registered study dietitian creates a nutrition plan individualised to the patient’s usual diet, offering the possibility of increasing food intake by adapting to the patient’s preferences, providing snacks and enriching/fortifying food. Furthermore, we provide the patients with an authorised and currently used high-energy, high-protein oral nutritional supplements (Nestlé Health Science, Switzerland) free of charge, as needed, to complement the nutritional strategy. Additionally, modular supplements such as maltodextrin or protein (Nestlé Health Science, Switzerland) may be employed if needed. To enhance adherence to nutritional therapy, the intervention group maintains regular contact with the study dietitian every 2–4 weeks. This approach allows for individualised adjustments to nutritional therapy based on patient preferences and enables close monitoring of food intake. If nutritional targets are not met, escalation to enteral or parenteral nutrition will be discussed with the study team and the treating physician. When the patient enters the acute terminal phase and no longer wishes to receive nutritional therapy, this is discontinued in accordance with ethical principles without noting any deviation from protocol. The nutritional algorithm used in EFFORTII is shown in figure 3.

In the control group, the study dietitian provides education on protein-rich and energy-rich diets at hospital discharge, but no additional oral nutritional supplements or nutritional counselling are offered to patients.

The trial includes the following visits:

- During hospitalisation: screening of patients, patient’s enrolment.
- Before hospital discharge: inclusion and randomisation, first visit of study dietitian.
- Every 2–4 weeks phone: phone call by unblinded study dietitian to monitor and potentially adjust nutritional intervention (intervention group only).
- At 3, 6 and 12 months: home visit (or in-clinic visits) by unblinded study dietitian to assess nutritional outcomes (both groups).
- Every 6 months: phone call follow-up for outcome assessment by blinded study nurse (both groups).

**Figure 4:**
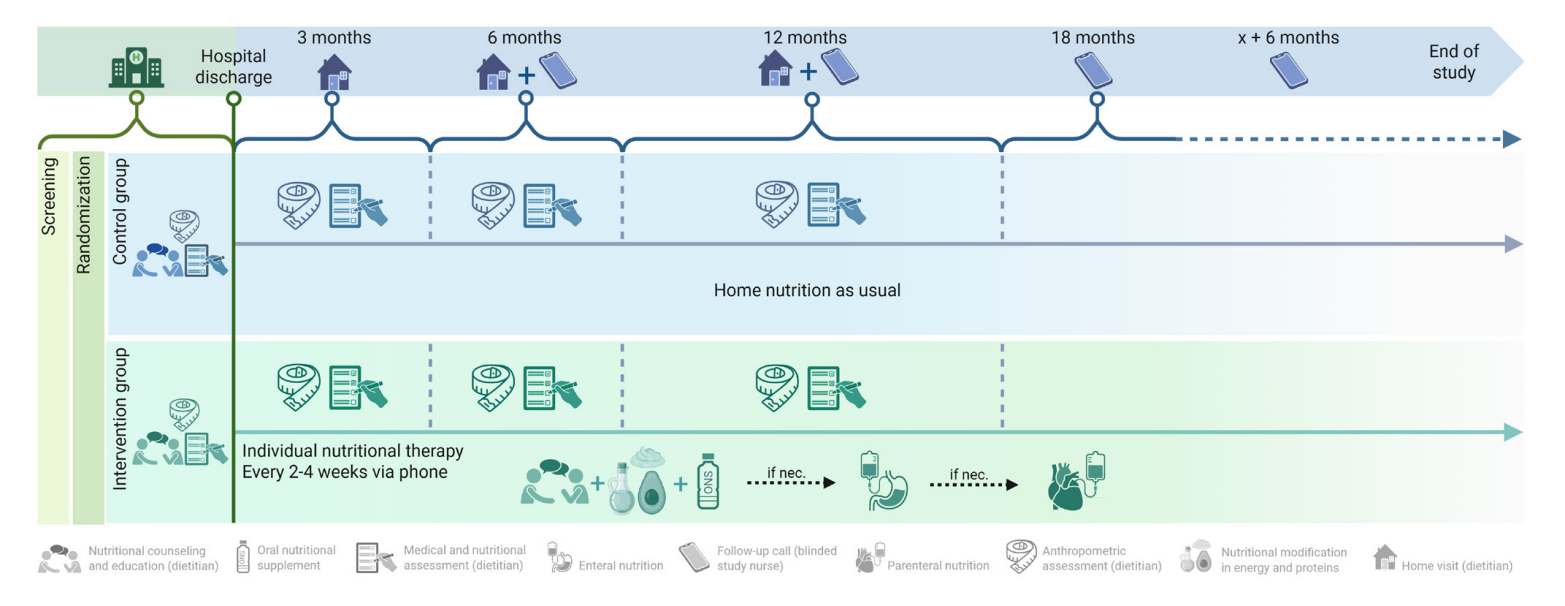
Overall patient flow.

Figure 4 shows the overall patient flow of EFFORTII.

## Ethics and dissemination

The study was approved by all local ethical committees of the participating hospitals. Written study agreements are obtained from all responsible head physicians and local principal investigators at each centre. All enrolled patients are asked to give written informed consent. Patients unable to provide informed consent are not included in the trial.

Importantly, despite the strong association between malnutrition and adverse clinical outcomes, assigning the control group to usual care is ethically justified by the principle of equipoise, given the uncertainty regarding the long-term effectiveness and safety of nutritional therapy in this patient population. This important subject has been discussed among national experts in the field (ie, collaborators) who all agree on this practice. This is also in accordance with a recent Swiss consensus ethical statement pointing out that intake of standard food and fluids is a basic right of any patient, yet any sort of nutritional therapy must be viewed as a therapeutic measure and must therefore fulfil all criteria for such including proof of clinical effectiveness, safety and cost-effectiveness.^31^ Because such evidence is lacking for this long-term care patient population, they represent the primary focus of this study. The Spanish study cohort recruits patients with conditions for which supplementation is not funded, such as COPD, heart failure or older adults with multiple comorbidities. Findings will be disseminated in peer-reviewed journals and academic conferences.

### Patient and public involvement statement

Patients were not directly involved in the initial design, recruitment or conduct of this RCT. However, patient representatives will actively be involved in the interpretation of the trial findings. A group of patient advisors, independent of the research team and representative of the target population, will be invited to participate in a results interpretation workshop after data analysis is completed. During this workshop, key findings will be reviewed and discussed from a patient perspective. Their feedback will be incorporated into the framing of the discussion section.

### Statistical approach

Detailed methodology for summaries and statistical analyses of the data collected in this study is documented in a statistical analysis plan, which will be finalised before database closure. The primary analysis population is the full analysis set, which, following intention-to-treat principles, includes all randomised patients. Every effort will be made to minimise the number of patients lost to follow-up. A secondary analysis population, the per-protocol (PP) population, will be prospectively defined to exclude patients with major protocol violations. Specifically, the following criteria will lead to exclusion from the PP population: major violation of study inclusion or study exclusion criteria, treatment not according to randomisation (eg, nutritional protocol not followed in a patient in the intervention group or control group patients receiving structured nutritional therapy) and patients lost to follow-up. A consort diagram will be reported as recommended.

The primary analysis will include only patients enrolled at the Swiss sites, as their recruitment and trial completion are expected to occur earlier due to the earlier trial start. After the Spanish cohort has also completed the study, a secondary publication is planned using a pooled, patient-level analysis.

For the primary endpoint, the time to death due to any cause, we will use a log-rank test to compare survival distributions in the control and intervention groups. To adjust for additional variables, we will fit a Cox proportional hazards model including age, Barthel’s index at baseline, study centre and initial NRS as covariates. For all variables included in the model, we will estimate the effect sizes and report 95% CIs. For the secondary endpoints, we will use survival analysis for all time-to-event outcomes and fit logistic and linear regression models for binary and continuous outcomes, respectively. Moreover, we will assess changes in functional outcomes and quality of life using a linear mixed model approach to account for the repeated measurements per patient. We will report effect estimates and CIs without p value adjustment for multiple comparisons. Additionally, we will analyse win-ratios to compare patient pairs across hierarchical clinical endpoints, determining a ‘win’ or ‘loss’ based on the most clinically important outcome within each pair.

We will perform predefined subgroup analyses by including interaction terms in the regression models to test effect modification by important baseline factors. Specifically, we will look at patient age (<60, 60–75, >75 years), sex, nutritional risk stratified by initial NRS (3, 4, >4 points), BMI (<20, 20–25, >25–30, >30 kg/m^2^), main medical diagnosis at the index hospital stay (systemic infection, heart failure, acute renal failure, gastrointestinal disease, tumour, COPD) and influential comorbidities (diabetes, chronic renal failure, status of inflammation) as well as socio-economic status (including food insecurity).

No interim analyses are planned.

### Sample size considerations

We designed the trial in collaboration with the Department of Clinical Research of University Hospital in Basel as an event-driven trial to show the superiority of continued nutritional therapy for patients discharged from the hospital compared with usual care regarding mortality. The sample size was calculated for the Swiss cohort only. We targeted 247 mortality events and a total sample size of at least 802 participants, who are assigned 1:1 to nutritional therapy and the usual care groups. We hypothesised an incidence of mortality of 20% per year in the usual care group, an effect estimate of 0.7, a type I error rate of 0.05, a power of 80%, a recruitment period of 1.5 years, a trial duration of 3 years and a withdrawal rate of 10% per year of follow-up.

**Figure 5:**
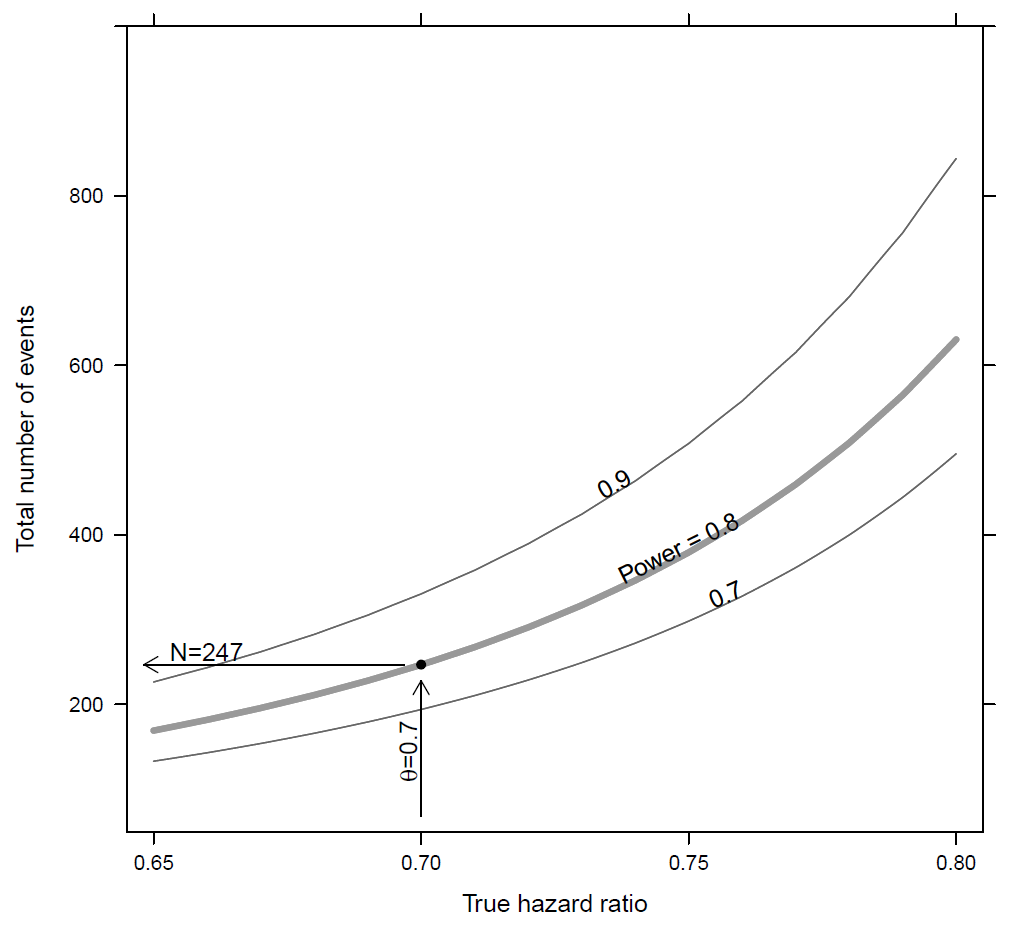
The number of events required is dependent on the true HR between the control and intervention groups. The required number of events for achieving a power of 80% as a function of the true HR. The curves for a power of 70% and 90% (ie, 0.7 and 0.9) are also shown.

Additionally, we calculated the required sample size for several different assumptions regarding the effect of our intervention, study durations and recruitment periods. Assuming an HR of only 0.75 and a power of 80%, the required sample size would be 1200. Thus, we recruit at least 802 participants but can continue to reach a maximal total of 1200 patients depending on timing/success of recruitment. Figure 5 demonstrates effect size estimations for the number of events needed in the trial.

For the Spanish cohort, a convenience sample of 180 patients, 20–40 patients in each of the six recruitment sites, was defined. While this sample size does not provide adequate power for within-cohort mortality analyses, its inclusion alongside the Swiss cohort will enhance the overall statistical power of the pooled analysis and may provide insights into aspects of external validity.

## Discussion

Disease-related malnutrition remains a highly prevalent and clinically significant concern among polymorbid medical patients, leading to increased morbidity, greater functional impairment and elevated mortality rates following hospital discharge.^2^ While prior inpatient trials such as EFFORT^32^ and NOURISH^18^ demonstrated that structured nutritional therapy can improve short-term outcomes, the sustainability of these effects beyond hospitalisation has remained uncertain. EFFORTII is designed to fill this knowledge gap by evaluating whether ongoing, personalised nutritional therapy after hospital discharge can lower long-term mortality and enhance functional recovery compared with usual care. We submit that EFFORTII will be the largest outpatient nutritional RCT to date and will provide robust evidence on whether medical patients at nutritional risk show a sustained benefit from long-term nutritional therapy after hospital discharge. Additionally, it is designed to elucidate the mechanisms by which such therapy influences the course of disease from a physiopathological perspective. Towards this aim, we have planned several secondary projects with the created data base focusing on mechanistic research questions. To support this objective, a dedicated biobank has been established as part of the clinical trial. Blood samples are collected at the main study centre in Aarau and stored for future analyses, enabling the identification and investigation of potential biomarkers. This resource will facilitate subsequent mechanistic research projects and allow emerging scientific insights to be explored through additional, later-stage analyses.

The strengths of this protocol include its pragmatic, multicentre, randomised design, its large sample size and its sequential two-cohort strategy, which allows both regional evaluation and pooled international estimates. By targeting multimorbid patients at nutritional risk, the trial focuses on a prevalent and high-risk group in which the potential for benefit is greatest. Furthermore, the comprehensive set of secondary outcomes, including readmissions, complications, functional capacity, quality of life and nutritional parameters, will provide a multidimensional assessment of the intervention’s impact. The inclusion of parameters that evaluate body and muscle composition (such as BIA and calf circumference), as well as muscle function (handgrip strength), functional status (Barthel Index) and quality of life (EQ-5D), may help identify patients at greater risk of complications and guide future personalised therapeutic strategies beyond body weight or BMI.^33^ Further, by incorporating pharmaco-economic research, EFFORTII will elucidate the indications in which nutritional therapy is cost-effective. Thus, EFFORTII will facilitate a more efficient healthcare resource distribution.

An important innovation of EFFORTII is the extension of individualised nutritional therapy into the vulnerable post-discharge period. This is a phase in which patients often experience persistent anorexia, inflammation and functional limitations, yet outpatient nutritional management remains fragmented and rarely standardised.^23^ If successful, EFFORTII would provide high-quality evidence to support updating current ESPEN^21^ and ASPEN^22^ guidelines, which currently offer only weak guidance for outpatient nutritional therapy due to the lack of large randomised evidence.

The EFFORT research team has a broad and established network of national and international partners and extensive experience in conducting nutrition RCTs. This ensures the quality of the implementation and the achievement of the recruitment target. Furthermore, the target group of polymorbid patients is likely to represent a large group of people, which enhances the generalisability of the results and reflects real-world clinical practice. Nevertheless, we are aware of certain limitations of this trial. First, due to the design of the intervention, it is not possible to blind patients or caregivers to their randomisation, which could lead to bias. In addition, bias may occur if caregivers feel compelled to motivate patients in the control group to eat more. Nevertheless, outcome assessments can be conducted in a blinded manner via structured telephone calls from a study nurse. Second, non-adherence to nutritional interventions in the outpatient treatment over a period of 1 year and longer is possible due to differences in motivation, comorbidities or social support, which may influence effectiveness. The trial mitigates this by frequent contact with the study dietitians and individual planning, but some residual non-adherence or lost-to-follow-up is likely.

In conclusion, this pragmatic research project is planned to improve the quality, effectiveness, safety and efficiency of nutritional therapy in the outpatient setting. It will also help us to better understand the relationship between nutrition and illness in the long term. Data acquired through EFFORTII will thus help healthcare professionals and payers worldwide to make better-informed decisions regarding the best care of polymorbid individuals after acute illness, who represent a large and growing patient population worldwide, and one that accounts for a major share of medical resource consumption.

## Supplementary material

10.1136/bmjopen-2025-115456 online supplemental file 1
