# Supplementary material for: Design and rationale of the EFFORTII project: a multicentric randomised-controlled trial on the impact of continued nutritional therapy at hospital discharge
Source: BMJ Open. 2026 Mar 18;16(3):e115456. doi: 10.1136/bmjopen-2025-115456 (PMC13007153; doi:10.1136/bmjopen-2025-115456)

**Supplemental Material**

**Protocol: Design and Rationale of the EFFORTII Project: A Multicentric Randomized-Controlled Trial on the Impact of Continued Nutritional Therapy at Hospital Discharge**

**Authors**

Carla Wunderle ^a^, Pascal Tribolet ^a, b, c,^, Nina Kaegi-Braun ^d^ , Valerie Haller ^a, e^, Robert Escher ^g^ , Drahomir Aujesky ^h^ , Gisele Trennepohl da Costa Heinen ^i^ , Michael Brändle ^j^ , Thomas Bregenzer ^k^ , Christoph Henzen ^l^ , Thomas Zehnder ^m^ , Susanne Schait ^n^ , Christina Gassmann ^o^ , Maja Dorfschmid ^p^ , Maria D Ballesteros-Pomar ^q^ , Cristina Cuerda ^r^ , Rosa Burgos ^s^, Daniel de Luis ^t,^ Gabriel Olveira ^u, v, w^ , Leocadio Rodriguez-Mañas ^x^ , Zeno Stanga ^h^, Beat Mueller ^a, f^ , Philipp Schuetz ^a, f^

^a^ Medical University Department, Division of General Internal and Emergency Medicine, Kantonsspital Aarau, Tellstrasse 25, 5001 Aarau, Switzerland

^b^ Department of Health Professions, Bern University of Applied Sciences, Murtenstrasse 10, 3008 Bern, Switzerland

^c^ Faculty of Life Sciences University of Vienna, Djerassiplatz 1, 1030 Vienna, Austria

^d^ Department of Medicine Huddinge, Karolinska Institute, 171 77 Stockholm, Sweden

^e^ Department of Nutritional Science, Justus Liebig University Giessen, Wilhelmstrasse 20, 35392, Giessen, Germany

^f^ Medical Faculty of the University of Basel, Klingelbergstrasse 61, 4056 Basel, Switzerland

^g^ Department of General Internal Medicine, Spital Emmental, Oberburgstrasse 54, 3400 Burgdorf, Switzerland

^h^ Division of Diabetes, Endocrinology, Nutritional Medicine, and Metabolism, Bern University Hospital and University of Bern, Freiburgstrasse 15, 3010 Bern, Switzerland

^i^ Department of Endocrinology, Kantonsspital Münsterlingen, Spitalcampus 1, 8596 Münsterlingen, Switzerland

^j^ Department of General Internal Medicine / Family Medicine and Emergency Medicine, HOCH Health Ostschweiz, Rorschacherstrasse 95, 9007 St. Gallen, Switzerland

^k^ Medical Department, Spital Lachen, Oberdorfstrasse 41, 8853 Lachen, Switzerland

^l^ Medical Department, Luzerner Kantonsspital, Spitalstrasse, 6000 Luzern 16, Switzerland

^m^ Medical Department, Spital Thun, Krankenhausstrasse 12, 3600 Thun, Switzerland

^n^ Department of Anesthesiology and Critical Care Medicine, Klinik Hirslanden, Witellikerstrasse 40, 8032 Zürich, Switzerland

^o^ Department of Nursing and Allied Health Care Professions, University Hospital Zurich, Universitätsspital Zürich, Rämistrasse 100, 8091 Zürich, Switzerland

^p^ Clinic for Visceral, Thoracic, Vascular Surgery, and Angiology, Stadtspital Zürich Triemli, Birmensdorferstrasse 497, 8063 Zürich, Switzerland

^q^ Department of Endocrinology and Nutrition, Complejo Asistencial Universitario de León, IBIOLEON, Calle Altos de Nava, s/n, 24008 León, Spain

^r^ Departamento de Medicina, Universidad Complutense de Madrid, Department of Endocrinology and Nutrition, Instituto de Investigación Sanitaria, Hospital General Universitario Gregorio Marañón, C. del Dr. Esquerdo, 46, Retiro, 28007 Madrid, Spain.

^s^ Nutritional Support Unit, University Hospital Vall d'Hebron, Pg. de la Vall d'Hebron, 119, Horta-Guinardó, 08035 Barcelona, Spain.

^t^ Servicio de Endocrinología y Nutrición, Hospital Clínico Universitario de Valladolid and Health Research Institute of Valladolid (IBioVALL., Av. Ramón y Cajal, 3, 47003 Valladolid, Spain. Centro de Investigacion de Endocrinología y Nutricion Fac Medicina Universidad de Valladolid. Centro de Investigación Biomedica en Red (CIBEROBN) de la Obesidad y Nutrición. Instituto de Salud Carlos III, Valladolid, Spain

^u^ Servicio de Endocrinología y Nutrición, Hospital Regional Universitario e instituto de investigación biomédica de Málaga/plataforma Bionand. Avd. Carlos Haya, 84, 29010 Málaga, Spain

^v^ Departamento de Medicina y Dermatología Universidad de Málaga, Spain

^w^ Centro de Investigación Biomédica en Red (CIBER) de Diabetes y Enfermedades Metabólicas Asociadas, Instituto de Salud Carlos III, Málaga, Spain

^x^ Centro de Investigación Biomédica en Red sobre Fragilidad y Envejecimiento Saludable (CIBERFES), Instituto de Salud Carlos III, Madrid, Spain; Servicio de Geriatría, Hospital Universitario de Getafe, Carr. Madrid - Toledo, Km 12,500, 28905 Getafe, Madrid, Spain

**Corresponding autor, prinicple investigator and sponsor:**

Philipp Schuetz, University Department of Medicine, Division of General Internal and Emergency Medicine, Kantonsspital Aarau AG, Tellstrasse 21, CH-5001 Aarau, Switzerland.

E-mail address: schuetzph@gmail.com

**sFigure 1: Patient Informed Consent Form (ICF) from main study site, Cantonal Hospital Aarau**


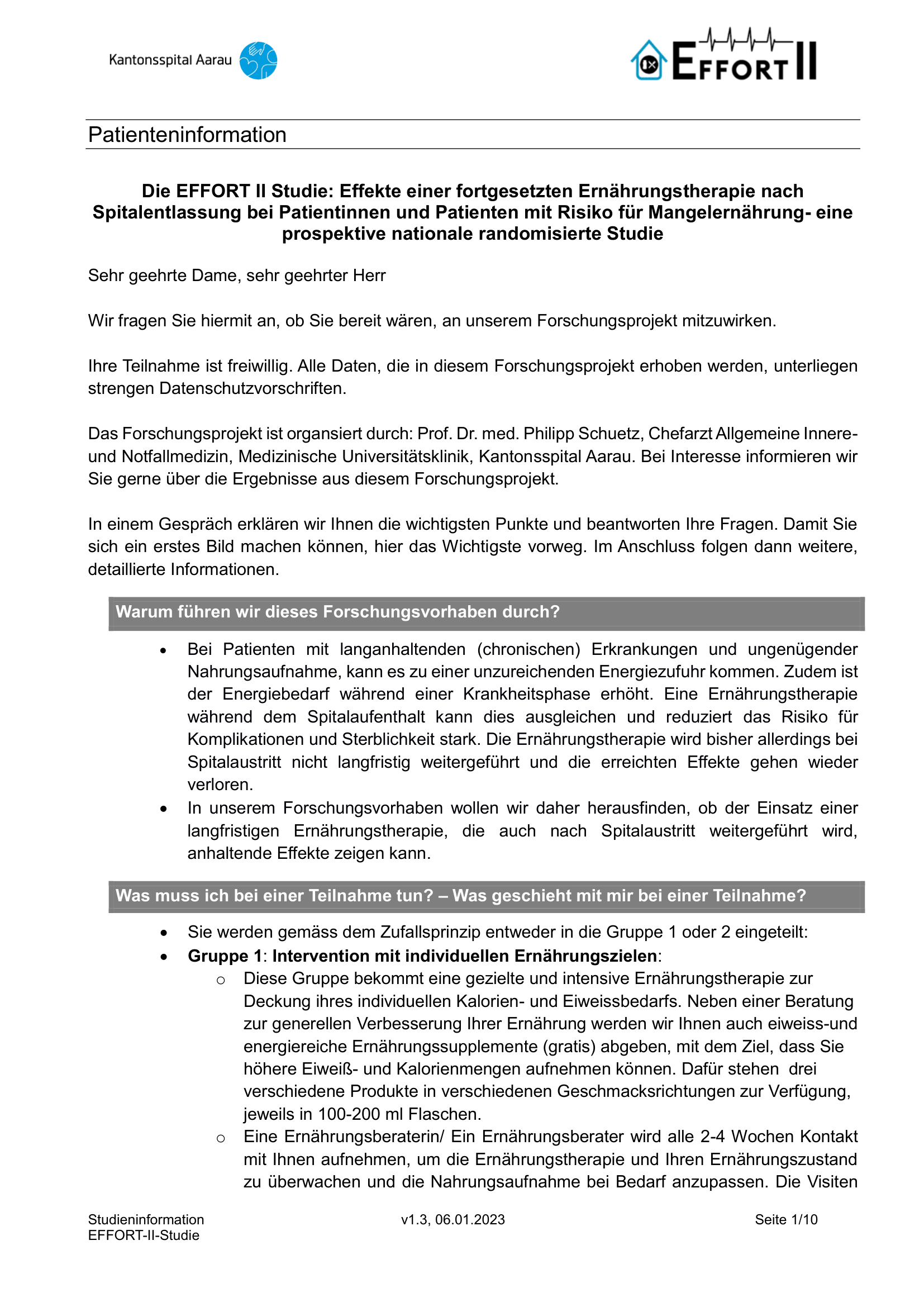

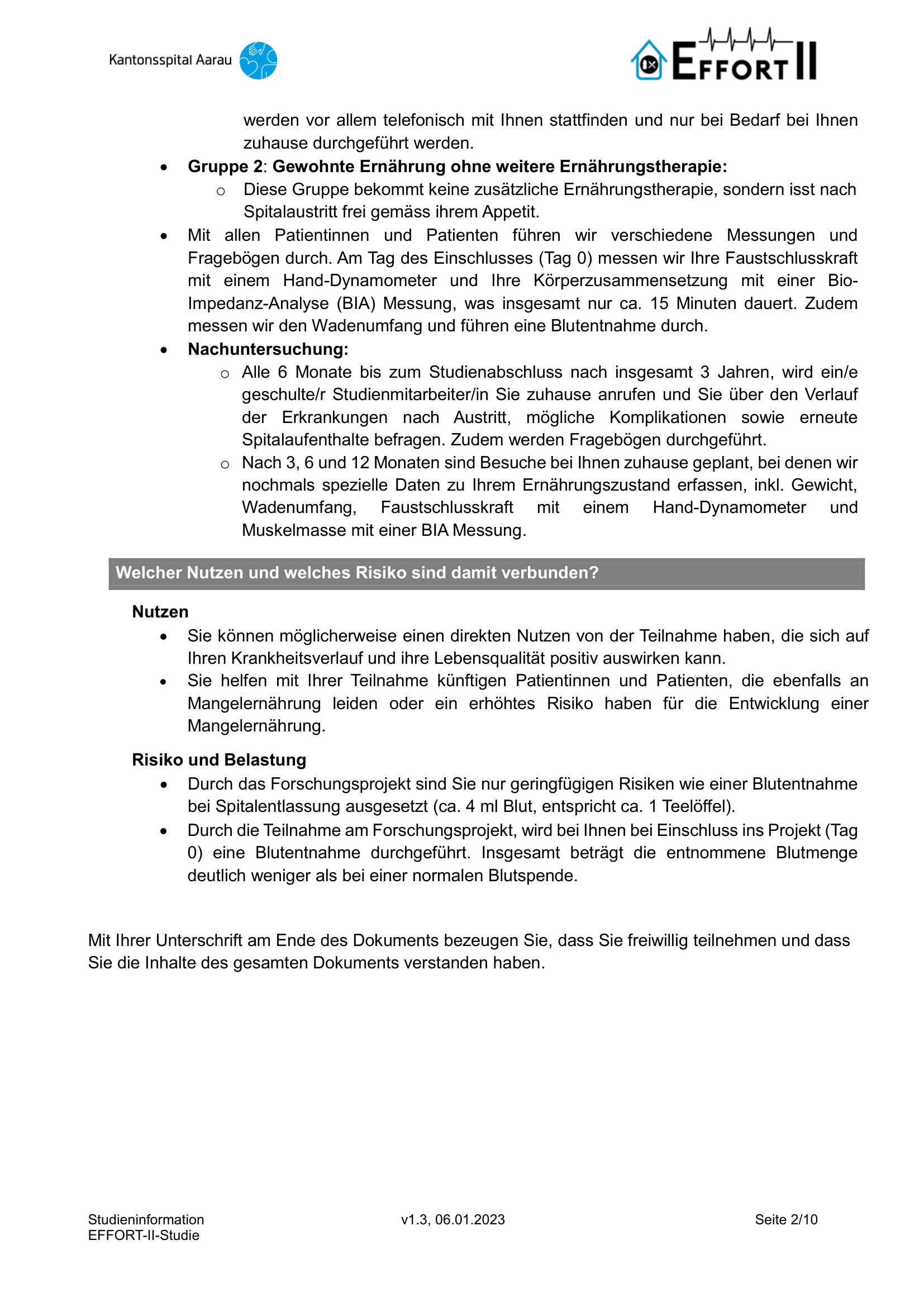

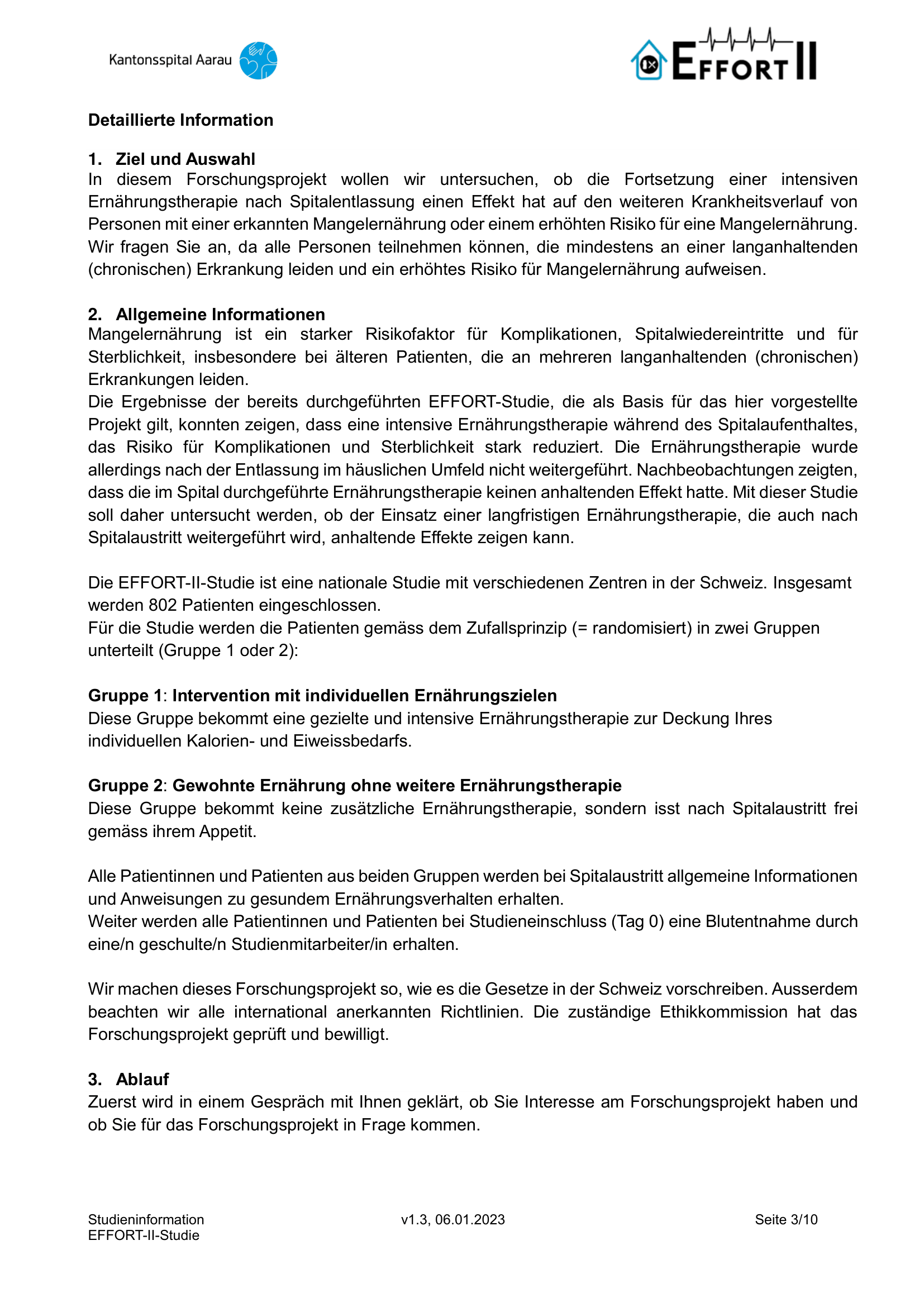

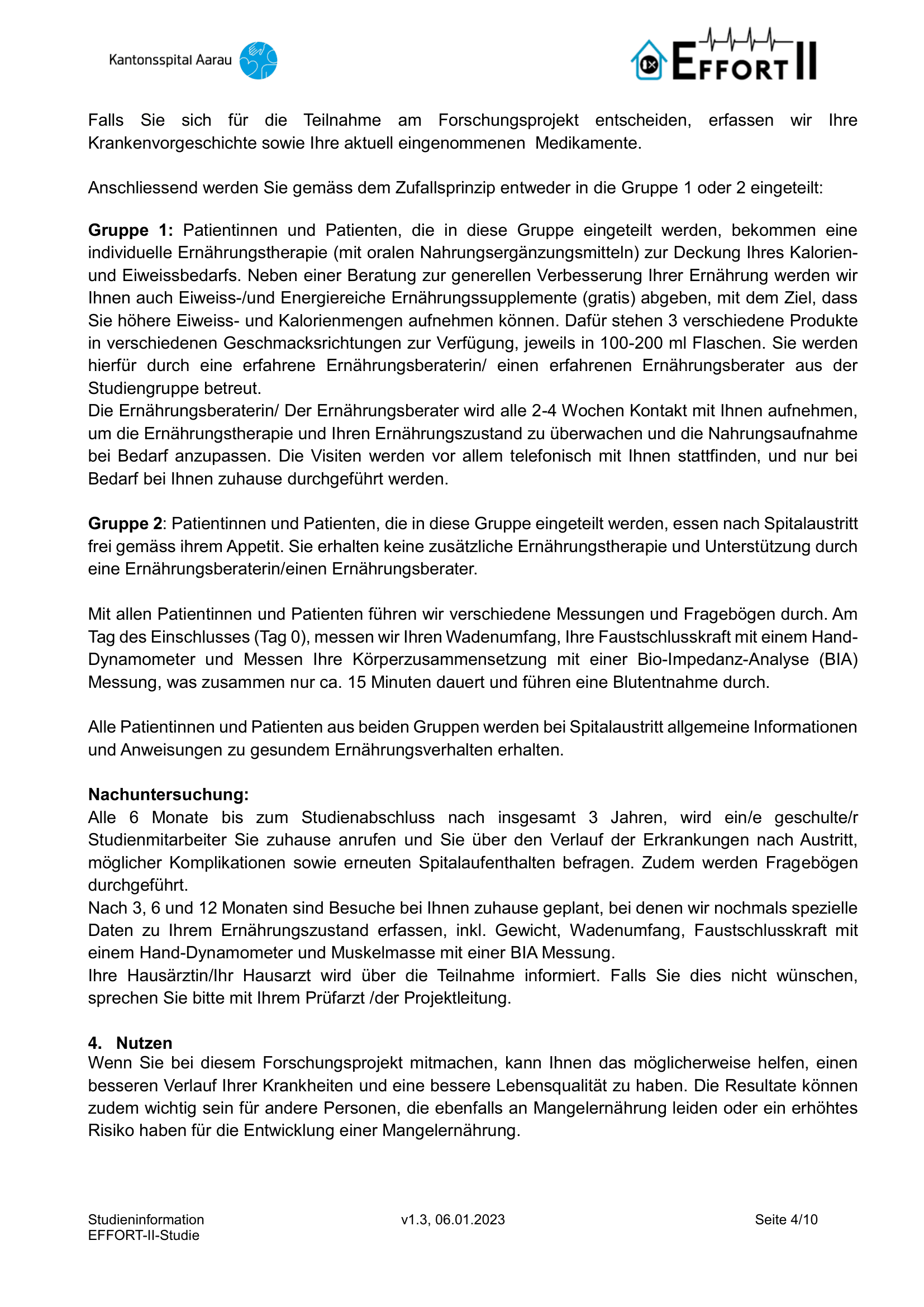

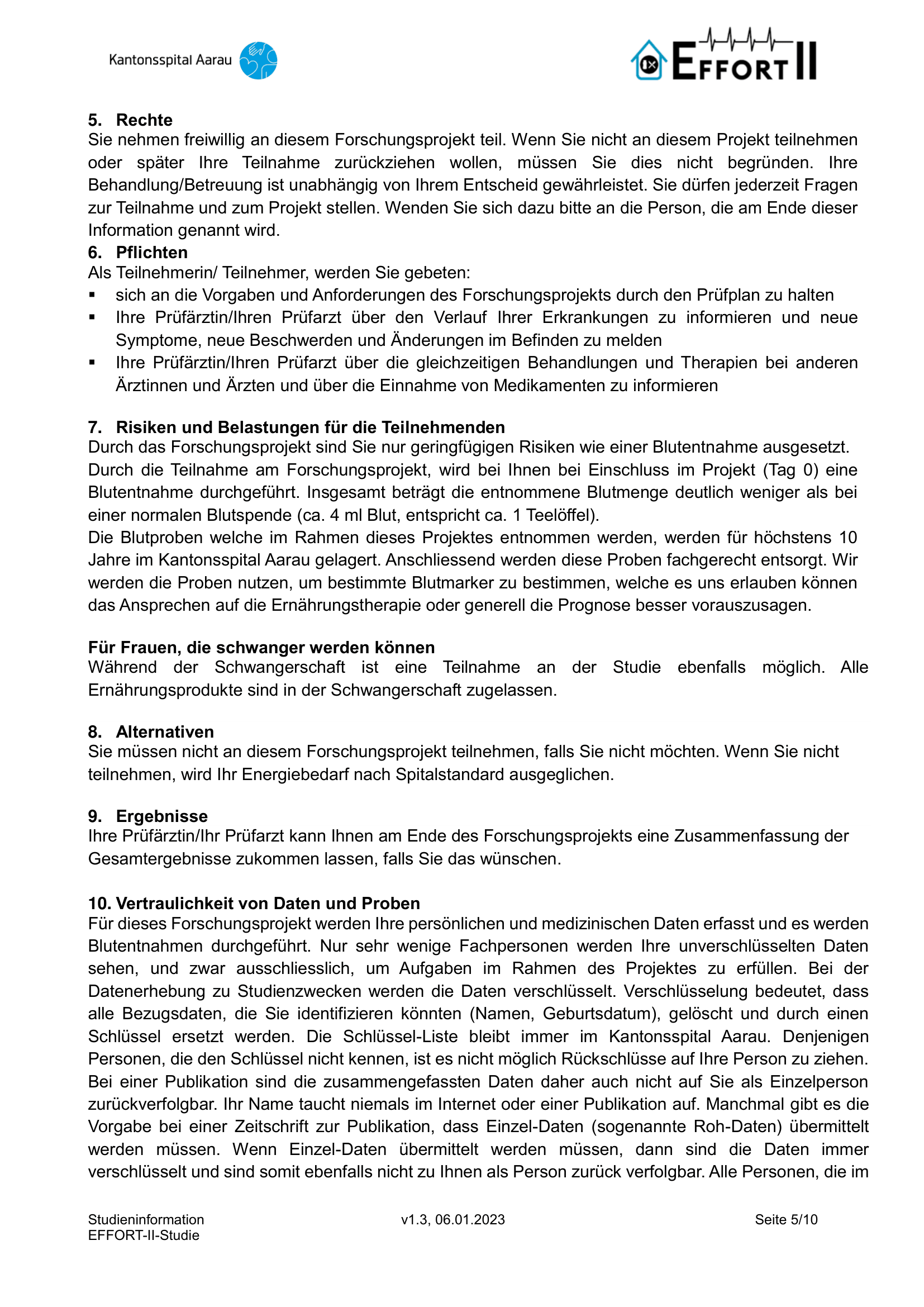

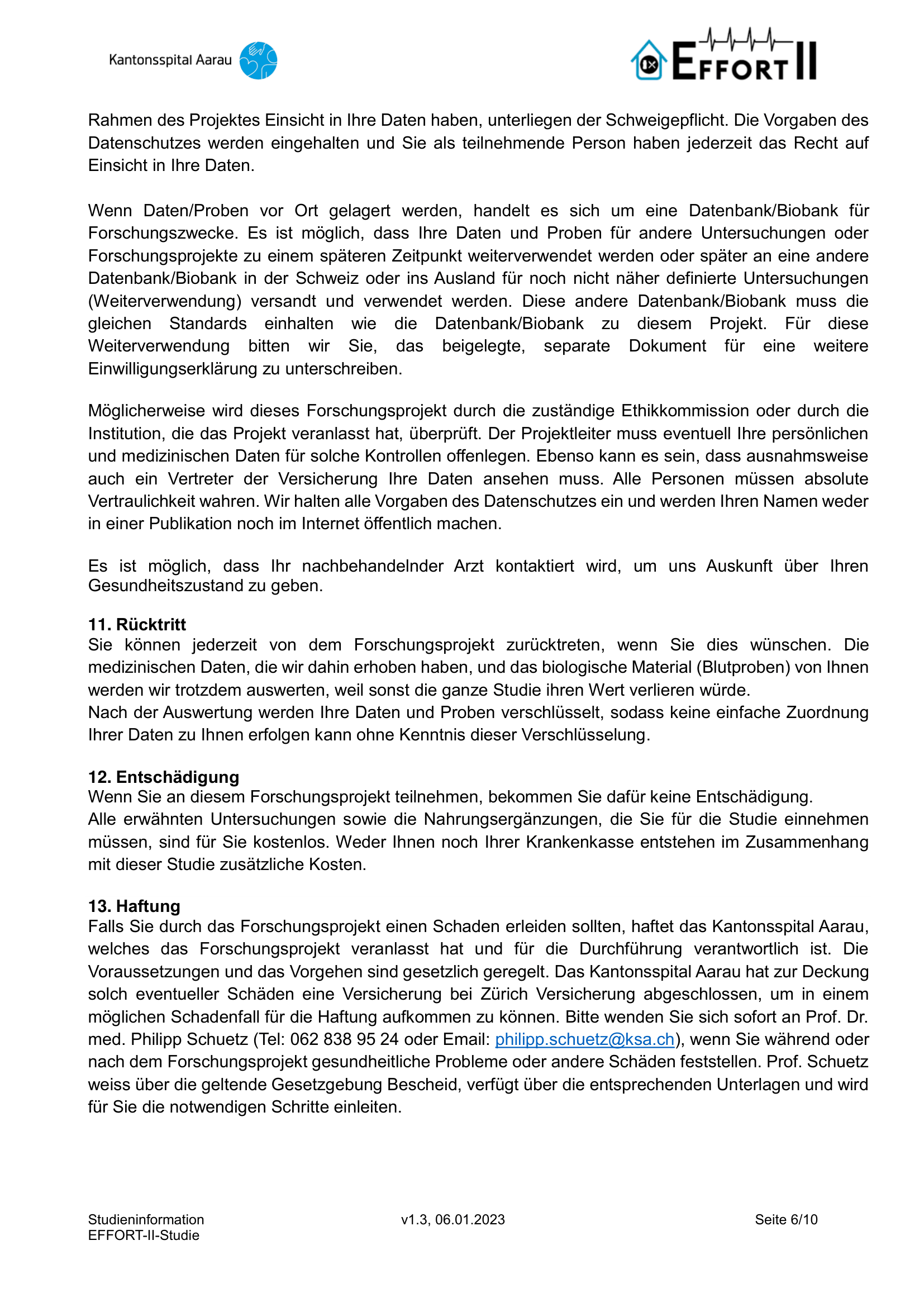

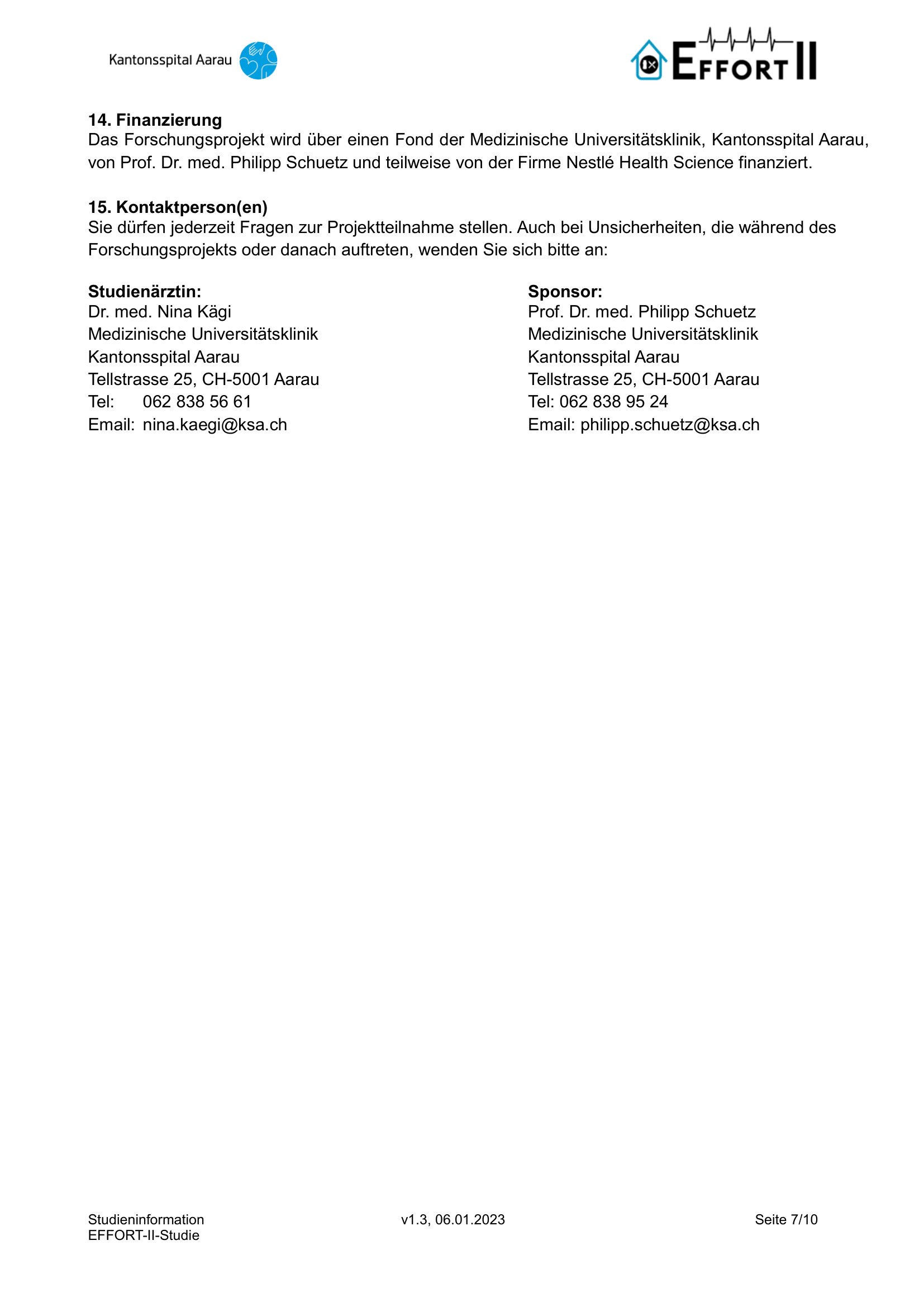

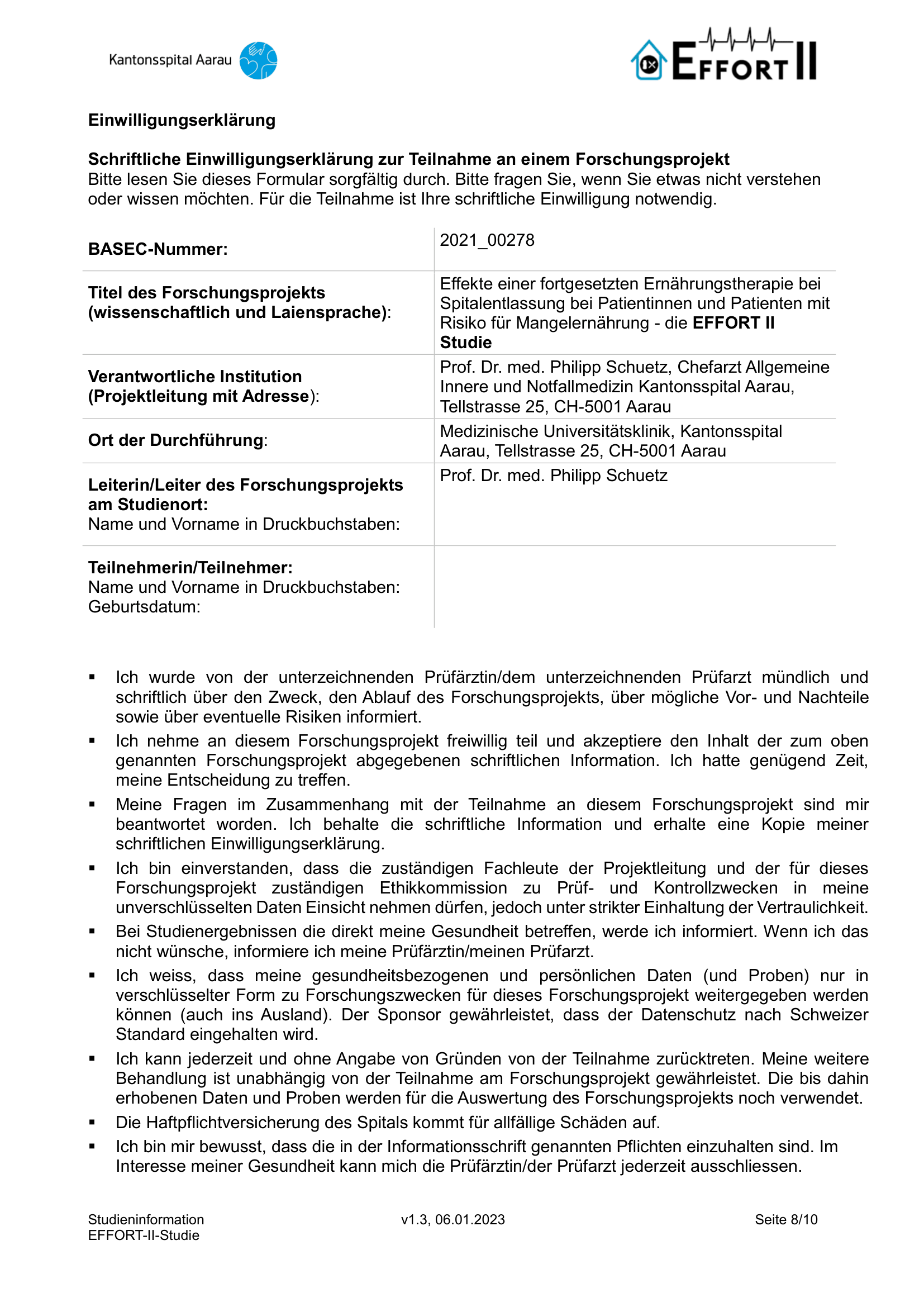

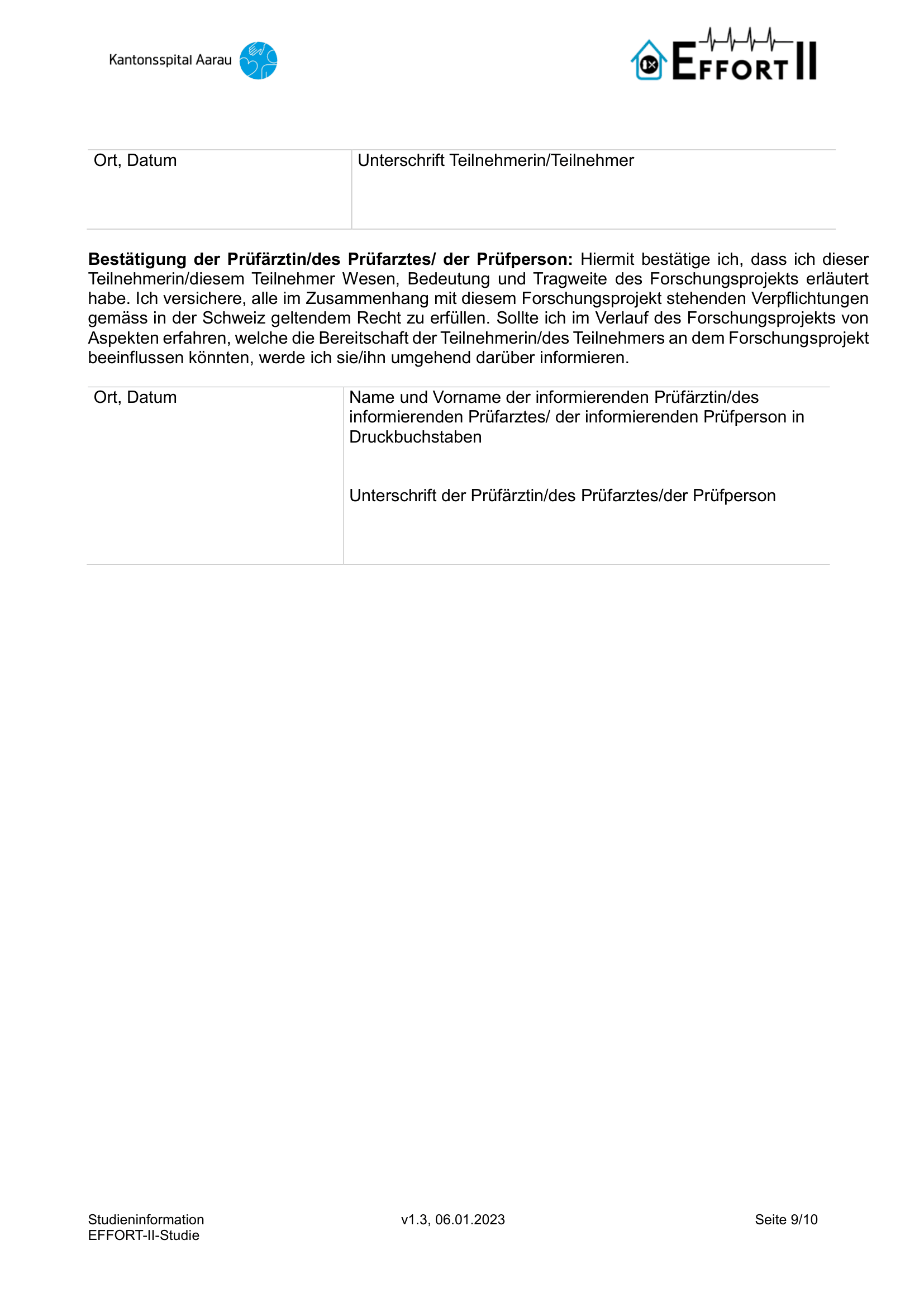

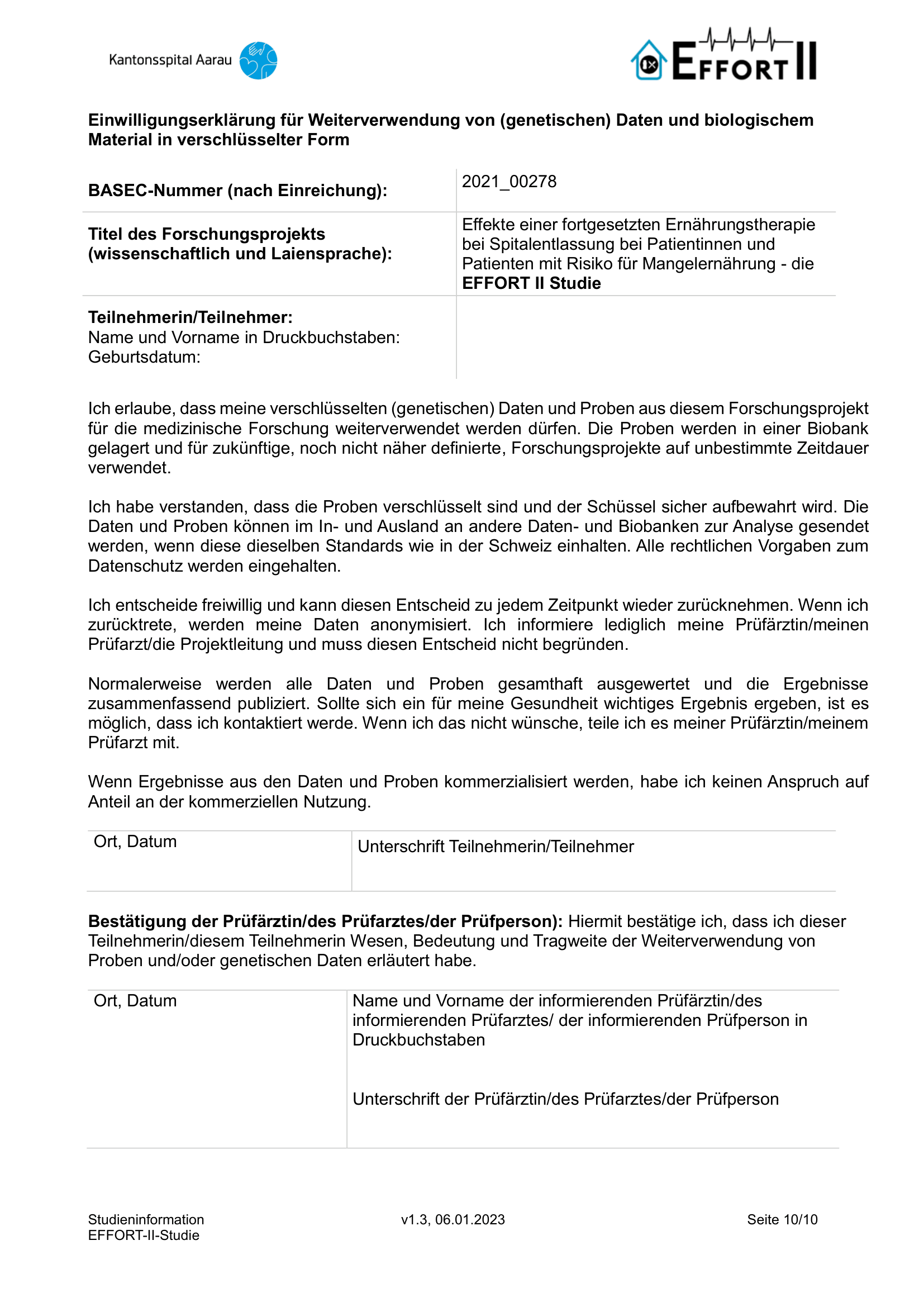

Supplement: online supplemental file 1 [file bmjopen-16-3-s001.docx]
