# Supplementary material for: Design and rationale of the EFFORTII project: a multicentric randomised-controlled trial on the impact of continued nutritional therapy at hospital discharge
Source: BMJ Open. 2026 Mar 18;16(3):e115456. doi: 10.1136/bmjopen-2025-115456 (PMC13007153; doi:10.1136/bmjopen-2025-115456)
Supplement: Supplementary file 2 [file bmjopen-2025-115456.reviewer_comments.pdf]

PEER REVIEW HISTORY

BMJ Open publishes all reviews undertaken for accepted manuscripts. Reviewers are asked to complete a checklist review form and are provided with free text boxes to elaborate on their assessment. These free text comments are reproduced below.

ARTICLE DETAILS

Title (Provisional)

Design and Rationale of the EFFORTII Project: A Multicentric Randomized-Controlled Trial on the Impact of Continued Nutritional Therapy at Hospital Discharge

Authors

Wunderle, Carla; Tribolet, Pascal; Kaegi-Braun, Nina; Haller, Valerie; Escher, Robert; AUJESKY , Drahomir; Trennepohl Da Costa Heinen, Gisele; Brändle, Michael; Bregenzer, Thomas; Henzen, Christoph; Zehnder, Thomas; Schait, Susanne; Gassmann, Christina; Dorfschmid, Maja; Ballesteros-Pomar, María; Cuerda, Cristina; Burgos, Rosa; De Luis, Daniel; Olveira, Gabriel; Rodriguez-Mañas, Leocardio; Stanga, Zeno; Mueller, Beat; Schuetz, Philipp

---

VERSION 1 - REVIEW

**Reviewer**	**1**
**Name**	**Morvaridi, Mehrnaz**
**Affiliation**	**Iran University of Medical Sciences**
**Date**	**03-Feb-2026**
**COI**	**None**

Thank you to the authors for submitting this protocol manuscript titled "Design and Rationale of the EFFORTII Project: A Multicentric Randomized-Controlled Trial on the Impact of Continued Nutritional Therapy at Hospital Discharge" to BMJ Open. Your work represents a significant contribution to the field of clinical nutrition, building on prior trials like EFFORT and addressing a critical evidence gap in post-discharge nutritional support for malnourished medical patients.

Detailed Comments

I have organized comments by section, focusing on scientific rigor, clarity, completeness, and adherence to protocol reporting standards. Page and line references are based on the provided manuscript.

Title and Structured Summary (Abstract)

Specify the follow-up duration for outcomes in the abstract (e.g., "assessed over up to 3 years or until event-driven endpoint met"). This would enhance clarity without exceeding word limits.

Introduction (Pages 5-8, Lines 112-175)

Briefly mention potential cost-effectiveness in the rationale (as it is a secondary focus), to tie into the methods section.

Methods (Pages 9-13, Lines 177-429)

Specify maximum follow-up duration (e.g., 3 years as in assumptions) to clarify trial end.

In endpoints, define "major complication" more precisely (e.g., acute renal failure criteria).

For statistics, mention multiple comparison adjustment if needed for secondaries (none stated, but conservative approach fine).

Ensure NutriOrg™ tool is briefly described or referenced.

Discussion (Pages 13-15, Lines 432-489)

Expand on how mechanistic ancillaries (e.g., biomarkers) will be analyzed/integrated.

VERSION 1 - AUTHOR RESPONSE

Thank you to the authors for submitting this protocol manuscript titled "Design and Rationale of the EFFORTII Project: A Multicentric Randomized-Controlled Trial on the Impact of Continued Nutritional Therapy at Hospital Discharge" to BMJ Open. Your work represents a significant contribution to the field of clinical nutrition, building on prior trials like EFFORT and addressing a critical evidence gap in post-discharge nutritional support for malnourished medical patients.
Detailed Comments
I have organized comments by section, focusing on scientific rigor, clarity, completeness, and adherence to protocol reporting standards. Page and line references are based on the provided manuscript.

Title and Structured Summary (Abstract)
Specify the follow-up duration for outcomes in the abstract (e.g., "assessed over up to 3 years or until event-driven endpoint met"). This would enhance clarity without exceeding word limits.
Reply: thank you for your comment. We have rephrased the abstract as suggested from line 68 as follows: "We designed the trial as an event-driven trial with a target of 247 mortality events (primary endpoint), which will be assessed over approximately 5 years until event-driven endpoint met. The minimum total sample size is at least 802 participants, based on the assumed treatment hazard ratio of 0.70."

Introduction (Pages 5-8, Lines 112-175)
Briefly mention potential cost-effectiveness in the rationale (as it is a secondary focus), to tie into the methods section.
Reply: thank you for your comment. We have added this rationale as follows from line 175: " Furthermore, we would like to clarify the indications for which nutritional therapy is cost-effective by incorporating pharmacoeconomic research."

Methods (Pages 9-13, Lines 177-429)
Specify maximum follow-up duration (e.g., 3 years as in assumptions) to clarify trial end.
Reply: thank you for your comment. We have specified study duration and termination from line 294 as

follows:
"We will follow participants until the end of the study, i.e., until approximately 247 mortality events have occurred. We expect a maximum follow-up period of 5 years for patients enrolled at the start of the study."

In endpoints, define "major complication" more precisely (e.g., acute renal failure criteria).
Reply: thank you for your suggestion. We assess major complications primarily through structured follow up calls and might confirm through a family member or the family physician if necessary. We describe it from line 274 as follows:
"All patients in both groups are contacted every 6 months by structured phone calls from a blinded study nurse or dietitian to assess primary and secondary endpoints until the patient reaches the primary endpoint, drops out or until the study is terminated. If necessary, information on patient mortality, or rehospitalizations or major complications during the follow-up period is confirmed through family members or the patient's primary care physician."

For statistics, mention multiple comparison adjustment if needed for secondaries (none stated, but conservative approach fine).
Reply: thank you for your comment. We will not adjust p-values for multiple comparisons, as it is not universally recommended. We will present effect estimates and confidence intervals, allowing readers to interpret the findings in context, which is also recommended by major methodological publications. We have clarified this from line 403 as follows:
"We will report effect estimates and confidence intervals without p-value adjustment for multiple comparisons."

Ensure NutriOrg™ tool is briefly described or referenced.
Reply: Thank you for your comment. We have added a description and the available reference from line 313 as follows:
"The nutritional intervention is supported by a web-based nutritional management system (NutriOrg™) developed by the EFFORT research team, which enables evidence-based, structured nutrition management. NutriOrg™ is a previously unpublished system that combines the previously publicly available tools from clinicalnutrition.science. (30)"
30. Huwiler VV, Tribolet P, Rimensberger C, Roten C, Schönenberger KA, Mühlebach S, et al. Implementation of evidence-based clinical nutrition: Usability of the new digital platform clinicalnutrition.science. Swiss medical weekly. 2025;155:3764.

Discussion (Pages 13-15, Lines 432-489)
Expand on how mechanistic ancillaries (e.g., biomarkers) will be analyzed/integrated.
Reply: Thank you for your comment. We have included your suggestion and rephrase the Discussion from line 450 as follows:
"Additionally, it is designed to elucidate the mechanisms by which such therapy influences the course of disease from a physio-pathological perspective. Towards this aim, we have planned several secondary projects with the created data base focusing on mechanistic research questions. To support this objective, a dedicated biobank has been established as part of the clinical trial. Blood samples are collected at the main study center in Aarau and stored for future analyses, enabling the identification and investigation of potential biomarkers. This resource will facilitate subsequent mechanistic research projects and allow emerging scientific insights to be explored through additional, later-stage analyses."
